# Prevalence and demographics of methicillin resistant *Staphylococcus aureus *in culturable skin and soft tissue infections in an urban emergency department

**DOI:** 10.1186/1471-227X-7-19

**Published:** 2007-10-31

**Authors:** Christian H Jacobus, Christopher J Lindsell, Sabrina D Leach, Gregory J Fermann, Amy Beth Kressel, Laura E Rue

**Affiliations:** 1 Department of Emergency Medicine, University of Cincinnati, Cincinnati, OH, USA; 2 Division of Infectious Diseases, Department of Medicine, Indiana University, Indianapolis, IN, USA; 3 University of Kentucky College of Medicine, Lexington, KY, USA

## Abstract

**Background:**

The rising incidence of methicillin resistant *Staph. aureus *(MRSA) infections is a concern for emergency practitioners. While studies have examined MRSA in inpatients, few have focused on emergency department populations. We sought to describe predictors of MRSA skin infections in an emergency department population.

**Methods:**

This was a prospective observational cohort study conducted over three months in 2005. A convenience sample of patients with culturable skin infections presenting to a busy, urban emergency department was enrolled. Demographic and risk factor information was collected by structured interview. The predictive value of each risk factor for MRSA, as identified by culture, was tested using univariable logistic regression, and a multivariable predictive model was developed.

**Results:**

Patients were 43% black, 40% female and mean age was 39 years (SD 14 years). Of the 182 patients with cultures, prevalence of MRSA was 58% (95%CI 50% to 65%). Significant predictors of MRSA were youth, lower body mass index, sexual contact in the past month, presence of an abscess cavity, spontaneous infection, and incarceration. The multivariable model had a C-statistic of 0.73 (95%CI 0.67 to 0.79) with four significant variables: age, group living, abscess cavity, and sexual contact within one month.

**Conclusion:**

In this population of emergency department patients, MRSA skin infection was related to youth, recent sexual contact, presence of abscess, low body mass index, spontaneity of infection, incarceration or contact with an inmate, and group home living.

## Background

Over the last decade, methicillin resistant *Staphylococcus aureus *(MRSA) skin infections have become increasingly prevalent in the emergency department (ED) [1,2]. While MRSA was historically limited to intensive care unit settings and to people in close contact with hospitals, there has been a recent increase in MRSA among people who have not had contact with the health care system [3]. As the number of patients with community-acquired MRSA grows, so does the need for emergency physicians to appropriately identify and treat MRSA infections.

### Importance

The prevalence of MRSA in the emergency department setting has been demonstrated as on the order of 60% in patients with culturable skin and soft tissue infections [1,2]. A recent report on MRSA prevalence concludes by suggesting physicians should provide antibiotics to cover the pathogen [2]. Such broad coverage might not be necessary if the physician can identify those patients at risk for MRSA. While various risk factors in the ED patient have been identified [1,2], the utility of such information in clinical practice has yet to be considered.

### Goals of this Investigation

We aim to further delineate risk factors for MRSA in the ED, and to develop a predictive model to help physicians identify those patients at risk for MRSA and those that may be treated more conservatively.

## Methods

### Theoretical model

The primary framework guiding our study is that MRSA is spread through skin-to-skin contact with infected individuals, unsanitary conditions, and activities placing individuals in proximity with infected persons. We therefore defined a set of variables we believed would increase the risk for MRSA, including demographics, BMI, homelessness, group or nursing home residence, incarceration, IV drug use, sexual contact, health care occupation, and recent skin infections. We additionally considered lesion characteristics as potential risk factors for MRSA (Table 1).

**Table 1:** Risk factors for MRSA

**Variable**	**Hypothesized relationship to MRSA**	**Found to be Predictive**
Age	Youth increases likelihood of engaging in other risky behaviors, i.e. drug use, sports, promiscuous sex	Yes
Race	Unknown, previously described in Frazee^1^	No
BMI	Elevated BMI suspected to impair ability to enact good hygiene	No
Homelessness	Suspected to impair ability to enact good hygiene	No
Group home	Increases skin to skin contact, communal living	Yes
Nursing home	Exposes one to hospital acquired pathogens	No
Incarceration, or contact with incarcerated person	Increases skin to skin contact, poor hygiene, transmission to family and friends	Yes
IV drug use	Inoculates bacteria directly into skin	No
Sexual contact	Increases skin to skin contact	Yes
Occupation in healthcare	Exposes to hospital acquired pathogens	No
Recent skin infection	Points to colonization with MRSA	No
Lesion characteristics (abscess/cellulitis)	MRSA tends to form abscesses or furuncles, rather than cellulitis^3^	Yes
Lesion location	Lesions in pelvic area lower risk given suspected higher incidence of fecal flora	No
Number of lesions	Increased number of lesions suspected to correlate with MRSA due to increased invasiveness of MRSA	No
Spontaneous vs. pre-existing wound	Spontaneous infections higher risk based on increased invasiveness of MRSA	No

### Study Design and Setting

This was a prospective observational cohort study of patients presenting to the ED of an urban tertiary care center with an annual census of 86,000 patients. The majority of patients at this ED are black (57%), and 67% receive Medicaid or are uninsured. The study was approved by the local institutional review board and an NIH certificate of confidentiality was obtained. The ED provides the local justice department with health care; prisoners were included in the sample with approval from the prisoner advocate.

### Selection of Participants

One investigator enrolled a convenience sample of eligible patients between August 2005 and October 2005. Patients with chief complaints of abscess, spider-bite, boil, cellulitis, or infection were targeted. Screening was by examination of triage notes, and by speaking to nurses and physicians. When a potential participant was identified, eligibility was confirmed by the treating physician through identification of a soft tissue or skin infection with culture collection being possible (i.e. spontaneous drainage or planned incision and drainage). Patients were excluded if they were under 18, if they were pregnant, if they presented to the ED on antibiotics appropriate for MRSA, if the abscess had already been drained and not cultured, or if the infection was an intra-oral or Bartholin's gland abscess (infections at low risk for MRSA) [4,5]. Patients of diminished mental capacity were included. If a patient had a legal guardian, the guardian provided consent, and the patient was asked to assent. All participants demonstrated capacity to consent by correctly answering five questions about the study after the consent document was read.

### Data Collection and Processing

A questionnaire was administered to all subjects that included questions about demographics as well as presence of suspected or known risk factors (Table 1) [6]. Race and ethnicity was self-reported. History of close contact with others with these risk factors was obtained. Height and weight were self reported or, if unknown, measured by the investigator. Characteristics of the infection (presence of a discrete collection of purulence, the presence of surrounding cellulitis, location of the lesions, and the number of lesions) as described by the treating physician were recorded.

When the treating physician obtained a culture, results of the culture were obtained from the patient's medical record when the culture was marked "final." If the treating physician did not order a wound culture, the investigator obtained an MRSA screening culture for study purposes only. The culture was obtained preferentially from the abscess cavity during incision and drainage, but also from topical draining purulence when no incision and drainage was performed.

Cultures were collected using a Becton-Dickinson BBL CultureSwab Plus collection and transport system (BD, Franklin Lakes, NJ) and sent immediately to the microbiology lab for processing. For all cultures S.*aureus *was identified by colony morphology, catalase, and the Pastorex Staph-Plus coagulase test (Bio-Rad Inc., Coquette-France). For cultures ordered by a treating physician, susceptibility testing was performed using the prompt inoculation system with MicroScan positive MIC panels following manufacturer's recommendations (MicroScan, Dade Behring Inc., West Sacramento, CA). Minimum inhibitory concentration (MIC) breakpoints and quality control were applied according to CLSI standards. For the investigator-ordered culture, methicillin resistance was detected using the Oxoid Penicillin Binding Protein (PBP2a) latex agglutination kit (Oxoid Limited, Besingstoke Hampshire, England) and only the presence or absence of MRSA or no growth was reported. Sensitivity and specificity of the investigator-ordered culture when compared to a reference MIC based method is 96.9 – 100% and 99.5 – 100%, respectively [7-9].

### Primary Data Analysis

Data are described using means and standard deviations or frequencies and proportions as appropriate. Univariable logistic regression was used to identify factors related to the presence or absence of MRSA. Variables significant at the p < 0.15 level were included in a multivariable logistic regression model, which was parsed manually based on a removal criterion of p < 0.1. Changes in the odds ratios, the Hosmer and Lemeshow goodness-of-fit, and the Akaike and Schwarze Information Criterion were considered; a large change in any one of these indicates the variable may be of importance regardless of statistical significance. The C-statistic was used as the global measure of model accuracy. The C-statistic is applied to the logistic regression model results in a similar way the Receiver Operating Characteristic (ROC) curve is applied to a diagnostic test result, and it is interpreted in the same way as the area under the ROC curve. A C-statistic of one indicates the model predicts MRSA perfectly, while a C-statistic of a half indicates the model predicts disease no better than chance. Analyses were conducted using SPSS v 13.0 (SPSS Inc., Chicago, Il).

## Results

Of 187 patients enrolled, five had no culture reported by the laboratory system and were excluded from the analysis. One eligible patient refused enrollment. Five cultures returned with no growth, these were considered negative for MRSA. Cultures with multiple pathogens were considered positive if any organism was MRSA. Patients are described in Table 2 stratified by MRSA positivity.

**Table 2:** Characteristics and risk factors for the patients enrolled in this study, stratified by the presence or absence of MRSA

	**No MRSA (N = 77)**		**MRSA (N = 105)**		**Total (N = 182)**	
Age	44.0	(15.2)	35.7	(11.6)	39.2	(13.8)
African American	31	(40.3)	48	(45.7)	79	(43.4)
Caucasian	46	(59.7)	57	(54.3)	103	(56.6)
Hispanic	1	(1.3)	4	(3.8)	5	(2.7)
Female	29	(37.7)	43	(41.0)	72	(39.6)
Male	48	(62.3)	62	(59.0)	110	(60.4)
BMI	30.5	(11.1)	27.1	(6.1)	28.6	(8.7)
**Sociodemographic risk factors**						
Homeless currently or in the past year	5	(6.5)	16	(15.2)	21	(11.5)
Live in a group home currently or in past year	3	(3.9)	13	(12.4)	16	(8.8)
Live in a nursing home currently	3	(3.9)	1	(1.0)	4	(2.2)
Prison resident currently or in past year	14	(18.2)	17	(16.2)	31	(17.0)
Participate in group sports in past year	14	(18.2)	23	(21.9)	37	(20.3)
Used IV drugs in past year	4	(5.2)	7	(6.7)	11	(6.0)
How many sex partners in past month	0.6	(0.7)	1.4	(2.7)	1.1	(2.1)
Healthcare worker currently or in past year	3	(3.9)	10	(9.5)	13	(7.1)
Sexuality						
Lesbian	1	(1.3)	0	(0.0)	1	(0.6)
Homosexual	2	(2.6)	3	(2.9)	5	(2.8)
Heterosexual	74	(96.1)	97	(93.3)	171	(94.5)
Bisexual	0	(0.0)	4	(3.8)	4	(2.2)
**Medical risk factors**						
Abscess, boil, spider bite or other requiring physician in past year	19	(24.7)	36	(34.3)	55	(30.2)
Diabetes	16	(20.8)	12	(11.4)	28	(15.4)
Antibiotics currently or in past year	33	(42.9)	48	(45.7)	81	(44.5)
Immunosuppressed	6	(7.8)	3	(2.9)	9	(4.9)
Inpatient in the past year	24	(32.9)	23	(23.5)	47	(27.5)
**Either been or been in contact with:**						
Homeless currently or in the past year	19	(24.7)	33	(31.4)	52	(28.6)
Live in a group home currently or in past year	14	(18.2)	27	(25.7)	41	(22.5)
Prison resident currently or in past year	21	(27.3)	45	(42.9)	66	(36.3)
Used IV drugs in past year	6	(7.8)	14	(13.3)	20	(11.0)
Healthcare worker currently or in past year	26	(33.8)	26	(24.8)	52	(28.6)
Diabetes	37	(48.1)	47	(44.8)	84	(46.2)
Abscess, boil, spider bite or other requiring physician in past year	26	(33.8)	48	(45.7)	74	(40.7)
**Infection**						
Abscess	54	(70.1)	96	(91.4)	150	(82.4)
Cellulitis	32	(41.6)	52	(49.5)	84	(46.2)
Infection on scalp or neck	4	(5.20	8	(7.6)	12	(6.6)
Infection on face	10	(13.0)	9	(8.6)	19	(10.4)
Infection on trunk	16	(20.8)	26	(24.8)	42	(23.1)
Infection on pelvis	12	(15.6)	21	(20.0)	33	(18.1)
Infection on Extremity	38	(49.4)	56	(53.3)	94	(51.6)
Number of infections	1.6	(3.0)	2.4	(3.6)	2.1	(3.3)
Spontaneous infection	43	(55.8)	80	(76.2)	123	(67.6)

**Figure 1:**
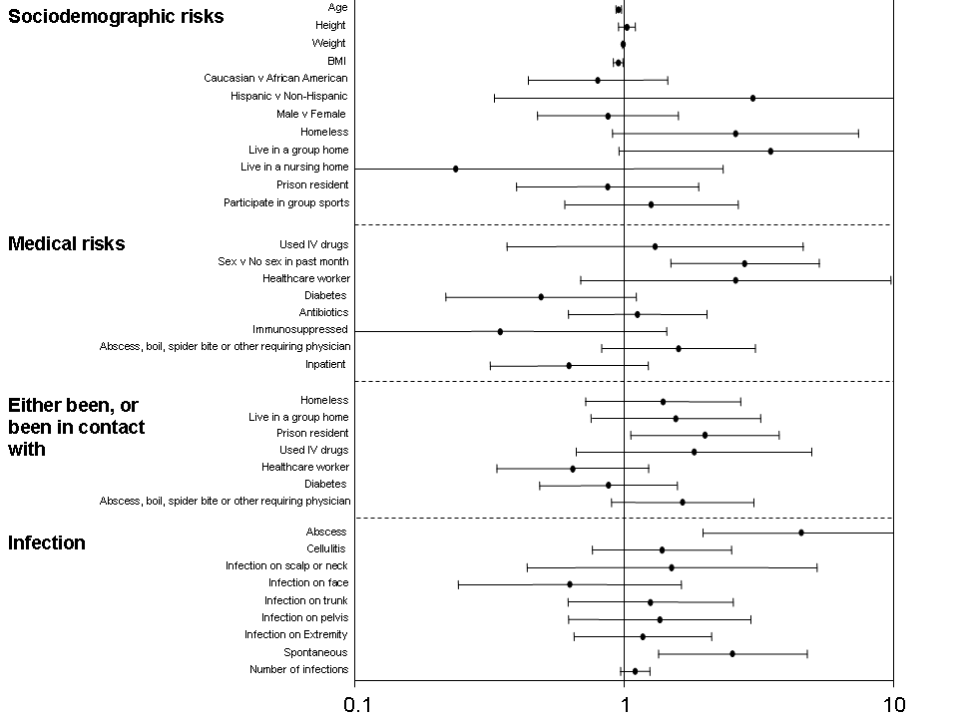
Odds ratios plus 95% confidence intervals for presence of MRSA.

There were 105 subjects (57.7%, 95%CI 50.4% – 64.6%) positive for MRSA. Increasing age, increasing weight and increasing BMI all decreased the odds of MRSA. A unit increase in each of these variables resulted in the following odds ratios: 1 year increase in age, OR 0.95 (95%CI 0.93–0.98); 1 pound (0.45 kg) increase in weight, OR 0.99 (95%CI 0.99 – 1.00); one unit (kg/m^2^) increase in BMI, OR 0.95 (95%CI 0.91 – 0.99). Factors increasing the odds of MRSA were sexual contact in the last month (OR 2.81; 95%CI 1.49 – 5.30), presence of a collection of purulence (OR 4.54; 95%CI of 1.96 – 10.50), spontaneous infection (versus a secondarily infected wound such as an abrasion or surgical wound) (OR 2.53; 95%CI 1.34 – 4.78), and history of incarceration within the last year or close contact with an inmate in the last month (OR 2.00; 95%CI 1.06 – 3.77) (Figure 1).

The best-fit, parsimonious multivariable model had a C-statistic of 0.73 (95%CI 0.67 to 0.79) with four predictor variables: increasing age decreased odds of MRSA (OR 0.97; 95%CI 0.95 – 1.00). Remaining factors increased odds of MRSA: group home residence (OR 4.71; 95%CI 1.17 – 18.90), presence of a discrete collection of purulence (OR 3.08; 95%CI 1.23 – 7.75), and sexual contact within the last month (OR 2.15; 95%CI 1.05 – 4.42) (Table 3). The C-statistic is a measure of how well a model can predict the presence of disease, with 1 being total accuracy and 0.5 being no better than chance.

**Table 3:** Multivariable logistic regression model to predict the presence of MRSA.

	**Odds ratio**	**95% CI (odds ratio)**	**p-value**
**Multivariable model**, C-statistic 0.765 (SE 0.036)			
Age	0.971	(0.943 – 1.001)	0.056
BMI	0.974	(0.932 – 1.018)	0.245
Homeless currently or in the past year	1.814	(0.506 – 6.498)	0.360
Prison resident or contact with prison resident currently or in past year	1.381	(0.666 – 2.865)	0.386
Live in a group home currently or in past year	3.827	(0.702 – 20.847)	0.121
Sex v No sex in past month	1.816	(0.835 – 3.950)	0.132
Diabetes	1.373	(0.459 – 4.103)	0.570
Immunosuppressed	0.573	(0.116 – 2.834)	0.495
Abscess, boil, spider bite or other requiring physician in past year, or contact with someone	1.255	(0.623 – 2.525)	0.525
Abscess	2.372	(0.831 – 6.769)	0.106
Spontaneous	1.890	(0.868 – 4.114)	0.109

**Parsimonious multivariable logistic model**, C-statistic 0.734 (SE 0.038)			
Age	0.971	(0.945 – 0.997)	0.032
Live in a group home currently or in past year	4.705	(1.173 – 18.871)	0.029
Abscess	3.084	(1.227 – 7.754)	0.017
Sex v No sex in past month	2.151	(1.047 – 4.422)	0.037

## Limitations

The primary limitation of this study is the convenience sample, which may have resulted in inclusion bias from two sources. First, enrollment in the study required an investigator to be present in the emergency department and second, it required the treating physician to confirm the presence of a skin abscess or infection. We have attenuated this latter source of bias by complementing routinely obtained cultures with a MRSA screening culture when the treating physician did not order a culture. Interestingly, only 10 screening cultures were needed, suggesting physicians routinely ordered cultures for skin and soft tissue infections. We mitigated the need for treating physician referral by routinely screening the emergency department population for likely study subjects during investigator hours. However, it was not possible to minimize bias associated with presentations when the investigators were not available. Thus, we cannot be sure that the study population is representative of the emergency department population presenting with infections.

Generalizability of our findings may be limited. Different communities have different rates of MRSA positivity, and perhaps different risk factors [2,6]. Thus results from this study conducted at a single ED may not extrapolate to other communities. Finally, our study was limited only to those infections in which a wound-site culture could be obtained. Conclusions from this study, therefore, should not be extrapolated beyond patients presenting to an Emergency Department with culturable skin infections.

## Discussion

There are few prior studies of MRSA skin infections in ED populations. Our prevalence of 58% is remarkably similar to the prevalences reported by Frazee^1 ^and Moran^2^, and reinforces the need for emergency practitioners to consider MRSA in all skin and soft tissue infections. Equally important, however, is the need to identify those at *low *risk for MRSA; as many antibiotics active against MRSA have poor coverage of other skin flora, empiric treatment of an unknown skin infection would require broad spectrum antibiotics. Pathogen specific treatment may help avoid the creation of further antibiotic resistance.

We studied commonly considered risk factors for MRSA infection: diabetes, incarceration, group or nursing home residence, homelessness, immunocompromise, team sport participation, intravenous drug use, sexual preference, occupation in healthcare, and recent antibiotic use [6]. Additionally, we considered BMI, sexual contact, close contact with individuals with risk factors, and physical descriptors of the infection as potentially related to MRSA. Similar to other ED-based studies [1,2], many of these commonly accepted risk factors for MRSA were not statistically associated with MRSA in our population. This may be due to a lack of statistical power; for a categorical predictor variable, our sample size would only achieve statistical significance with 80% power if the odds ratio were relatively large, greater than about 2.5, assuming the risk factor occurred about 50% of the time in one of the two groups. Alternatively, differences between underlying study populations may explain why risks among ED patients appear to differ, perhaps because the ratio of community acquired MRSA to hospital acquired MRSA is different in the ED patients.

Age has been previously described as a risk factor for community acquired MRSA [10]. While we did not distinguish between hospital and community acquired MRSA we did find a trend towards MRSA among younger subjects, which we hypothesize to be due to most of the MRSA in our study being community acquired. The decreased risk for MRSA with increasing age is small, but additive. The difference in risk between an 18 and a 70-year-old patient with no other risk factors is 37% versus 10%. The risk among younger patients is likely related to increased participation in risky activities such as team sports rather than physiologic changes due to aging. Further studies to verify or refute this finding are warranted.

MRSA tends to be a socially transmitted organism and the majority of risk factors appear to be inter-personal interactions. As such, we raised the question of whether MRSA could be "sexually transmitted." Sexual contact as an MRSA risk factor has only recently been described [11]. We originally hypothesized that sex workers would be at increased risk for MRSA due to increased skin-to-skin contact. To avoid ethical concerns arising from asking about the exchange of sex for money, we asked for number of recent sexual partners as a surrogate. We did not show a discernable difference in risk between a patient with only one partner and a patient with multiple partners, suggesting the occurrence of sexual contact and not the frequency of sexual contact is of greatest import. Physicians might benefit from obtaining a sexual history from patients with skin and soft tissue infections prior to ascertaining which antibiotics to prescribe, if any.

Recent data suggests that the majority of MRSA infected patients get better no matter which antibiotic they are prescribed [2], which suggests perhaps MRSA need not be identified. We contend that broad spectrum coverage for every skin infection that warrants antibiotics may hasten further drug resistance. As many of the antibiotics active against MRSA have poor coverage for other typical skin flora, risk stratification for MRSA remains important. While a mathematical prediction rule to identify high risk for MRSA positivity in an infection can be based on our multivariable model, clinically relevant diagnostic information can also be derived. For example, the likelihood ratio is 0.3 for MRSA for a patient older than 24 years, who does not live in a group home, has not had sex in the last month, and does not have a discrete collection of purulence. In other words, our data would suggest that meeting these criteria decreases the odds of disease to about 1/3 of the pre-test odds.

## Conclusion

Our results suggest that MRSA is present in over half of the skin infections presenting to our urban emergency department. Age less than 24, group home residence, the presence of an abscess cavity, and sexual activity within the last month should prompt the emergency physician to increase their suspicion for MRSA, while the absence of all of these factors should decrease suspicion for MRSA.

## Competing interests

The author(s) declare that they have no competing interests.

## Authors' contributions

This study was conceived by CJ and designed by CJ, SL, GF, LR, AK and CJL. CJ was responsible for obtaining funding and data collection. CJL conducted the statistical analysis, CJ and CJL were responsible for interpreting the data. All authors provided critical review of the manuscript. CJ takes full responsibility for the integrity of the data and for the manuscript as a whole.

## Pre-publication history

The pre-publication history for this paper can be accessed here:



## References

[B1] Frazee BW, Lynn J, Charlebois ED, Lambert L, Lowery D, Perdreau-Remington F (2005). High prevalence of methicillin-resistant Staphylococcus aureus in emergency department skin and soft tissue infections. Annals of Emergency Medicine.

[B2] Moran GJ, Krishnadasan A, Gorwitz RJ, Foshein GE, McDougal LK, Carey RB, Talan DA (2006). Methicillin-Resistant *S. aureus *Infections among Patients in the Emergency Department. NEJM.

[B3] Herold BC, Immergluck LC, Maranan MC, Lauderdale DS, Gaskin RE, Boyle-Vavra S, Leitch CD, Daum RS (1998). Community-acquired methicillin-resistant Staphylococcus aureus in children with no identified predisposing risk. JAMA.

[B4] Kuriyama T, Karasawa T, Nakagawa K, Saiki Y, Yamamoto E, Nakamura S (2000). Bacteriologic features and antimicrobial susceptibility in isolates from orofacial odontogenic infections. Oral Surgery, Oral Medicine, Oral Pathology, Oral Radiology, and Endodontics.

[B5] Tanaka K, Mikamo H, Ninomiya M, Izumi T, Ito K, Yamaoka K, Watanabe K (2005). Microbiology of Bartholin's gland abscesses in Japan. Journal of Clinical Microbiology.

[B6] Salgado CD, Farr BM, Calfee DP (2003). Community-Acquired Methicillin-Resistant *Staphylococcus aureus: *A Meta-Analysis of Prevalence and Risk Factors. Clinical Infectious Diseases.

[B7] Louie L, Matsumura SO, Choi E, Louie M, Simor AE (2000). Evaluation of Three Rapid Methods of Detection of Methicillin Resistance in Staphylococcus aureus. J Clin Microbiol.

[B8] Hussain Z, Stoakes L, Garrow S, Longo S, Fitzgerald V, Lannigan R (2000). Rapid Detection of *mecA*-positive and *mecA*-negative Coagulase-Negative Staphylococci by an Anti-Penicillin Binding Protein 2a Slide Latex Agglutination Test. J Clin Microbiol.

[B9] Yamazumi T, Marshall SA, Wilke WW, Diekema DJ, Pfaller MA, Jones RN (2000). Comparison of the Vitek Gram-Positive Susceptibility 106 Card and the MRSA-Screen Latex Agglutination Test for Determining Oxacillin Resistance in Clinical Bloodstream Isolates of *Staphylococcus aureus*. J Clin Microbiol.

[B10] Naimi TS, Ledell KH, Como-Sabetti K, Borchardt SM, Boxrud DJ, Etienne J, Johnson SK, Vandanesch F, Fridkin S, O'Boyle C, Danila RN, Lynfield R (2003). Comparison of Community – and Health Care-Associated Methicillin-Resistant *Staphylococcus aureus *Infection. JAMA.

[B11] Cook HA, Furuya EY, Larson E, Vasquez G, Lowy FD (2007). Heterosexual Transmission of Community-Associated Methicillin-Resistant *Staphylococcus aureus*. Clinical Infectious Diseases.

